# Ketogenic diet impairs neurological development of neonatal rats and affects biochemical composition of maternal brains: evidence of functional recovery in pups

**DOI:** 10.1007/s00429-021-02450-1

**Published:** 2022-01-17

**Authors:** Wojciech Kosiek, Zuzanna Rauk, Piotr Szulc, Anna Cichy, Marzena Rugieł, Joanna Chwiej, Krzysztof Janeczko, Zuzanna Setkowicz

**Affiliations:** 1 Laboratory of Experimental Neuropathology, Institute of Zoology and Biomedical Research, Faculty of Biology, Gronostajowa 9, 30-387 Kraków, Poland; 2 Faculty of Biochemistry, Biophysics and Biotechnology, Gronostajowa 7, 30-387 Kraków, Poland; 3 Faculty of Physics and Applied Computer Science, AGH University of Science and Technology, 30-059 Krakow, Poland

**Keywords:** Ketosis, Fetal growth, Undernutrition, Betahydroxybutyrate, FTIR spectroscopy

## Abstract

The ketogenic diet (KD) is a type of diet in which the intake of fats significantly increases at the cost of carbohydrates while maintaining an adequate amount of proteins. This kind of diet has been successfully used in clinical therapies of drug-resistant epilepsy, but there is still insufficient evidence on its safety when used in pregnancy. To assess KD effects on the course of gestation and fetal development, pregnant females were fed with: (i) KD during pregnancy and lactation periods (KD group), (ii) KD during pregnancy replaced with ND from the day 2 postpartum (KDND group) and (iii) normal diet alone (ND group). The body mass, ketone and glucose blood levels, and food intake were monitored. In brains of KD-fed females, FTIR biochemical analyses revealed increased concentrations of lipids and ketone groups containing molecules. In offspring of these females, significant reduction of the body mass and delays in neurological development were detected. However, replacement of KD with ND in these females at the beginning of lactation period led to regainment of the body mass in their pups as early as on the postnatal day 14. Moreover, the vast majority of our neurological tests detected functional recovery up to the normal level. It could be concluded that the ketogenic diet undoubtedly affects the brain of pregnant females and impairs the somatic and neurological development of their offspring. However, early postnatal withdrawal of this diet may initiate compensatory processes and considerable functional restitution of the nervous system based on still unrecognized mechanisms.

## Introduction

A ketogenic diet is a type of low-carbohydrate diet with moderate protein restriction and no restriction on fats as a result of which leading the body into a state of ketosis (Kirkpatrick et al. 2019; Gzieło et al. 2020). During a ketogenic diet, fat intake should be around 80% of total caloric consumption, however, there are many other variations with different nutrient distribution.

Under normal circumstances, energy required to maintain a proper functional status in the adult brain derives from glucose generated in carbohydrate metabolism. When carbohydrates are eliminated from the diet, the energy comes from fatty acid oxidation generating the amount of acetyl-Co molecules sufficient for the synthesis of acetylacetic acid in the liver. It can be spontaneously transformed into acetone or enzymatically transformed into beta-hydroxybutyric acid (ketone bodies, BHB). This reaction can run both directions in the presence of beta-hydroxybutyric acid dehydrogenase (Bailey et al. 2005). Ketone bodies generated in this way become an alternative energy source. The condition in which the synthesis of ketone bodies is accelerated is called ketosis. Ketone bodies are transported to the circulation and then to extrahepatic tissues where they are used as ATP precursors. They can also reach the brain easily crossing the blood–brain barrier (acetone via simple diffusion, beta-hydroxybutyric acid and acetylacetic acid through appropriate transporters), the expression of which is connected with the ketosis level (Bailey et al. 2005). Upon crossing the blood brain barrier, ketones are transported across cell plasma membranes via MCTs 1 and 2 in astrocytes and neurons, respectively (Vijay and Morris 2014).

Currently, due to the increase in popularity of the diet, it is used by bodybuilders during downsizing or as a general nutritional model among adults although it was originally developed to treat epilepsy, nervous system disorders, and obesity (Lee and Lee 2021a, b; Zhang et al. 2018). The increased interest in dieting especially among women during their childbearing period raises legitimate concerns because this dietary style may affect the developing fetus, its nervous system and can influence the maternal nervous system as well.

Maternal nutrition during pregnancy is an important part of maternal and child health care and determines what and how much of what passes through the placenta to the fetus (Jansson and Powell 2007). Changes in diet can have detrimental effects on the metabolism of both mother and child (King 2000). A key aspect is caloric intake, which directly affects fetal growth and, more specifically, body mass gain during pregnancy. During the whole pregnancy period, energy requirements gradually increase but within the first trimester they still remain at the normal level (Williamson 2006; Mousa et al. 2019). In mice on a ketogenic diet, due to the fetus access to the high-energy fuel ketone bodies, there is a rapid volume gain at the early stage of pregnancy with, however, a subsequent decrease (Sussman et al. 2013). Proteins are important nutrients due to their structural and functional role. To maintain homeostasis during pregnancy, maternal adaptation is necessary to the increased demands for these macronutrients resulting from fetal requirements and preparation for lactation (Elango and Ball 2016). However, it should be noted that both low-protein and high-protein diets are associated with detrimental effects on the offspring and may lead to the development of various diseases at a later stage (Langley-Evans 2009). In mouse embryos on a high-protein diet, an increased amount of reactive oxygen species can be seen while on a low-protein diet there is a reduced mitochondrial count. This indicates that, in the case of protein, it affects the intensity of metabolic processes (Mitchell et al. 2009). Although all nutrients have influence on brain maturation, proteins appear to be the most critical components for the development of neurological functions. Experimental studies in rodents have shown that perinatal protein malnutrition could alter neurogenesis, cell maturation, synaptic plasticity and physical and neuromuscular development (Ferroni et al. 2020).

In the case of carbohydrates, their main influence comes from being the main energy substrate and their intake should correlate with the increase in demand during pregnancy. However, attention should be paid to the glycemic index of the food consumed, as its high value leads to placental hypertrophy and excessive maternal body mass gain (Clapp 2002). Fats are important nutrients in the later stages of pregnancy, during the development of the nervous system. It has been noted that fat intake can affect the later behavior of children (Gustafsson et al. 2016). Preclinical models show that DHA (Docosahexaenoic acid) is necessary for neurogenesis and neuronal migration, membrane fatty acid composition and fluidity, and synaptogenesis. The LC-PUFAs have a profound effect on monoaminergic, cholinergic, and GABA-ergic neurotransmitter systems (Cusick and Georgieff 2016).

Due to the numerous reports related to fetal development and neurogenesis during ketogenic diet therapy, we examined how the ketogenic diet, applied during pregnancy and lactation periods, affects the biochemical composition of maternal brain and neurological development of pups originating from the affected females. If this diet were to have negative consequences, it would be very important to establish whether they are irreversible, or if discontinuation of this diet would allow for a general somatic and functional recovery.

## Materials and methods

### Animal treatment in pregnancy and postpartum

In the experiment, we used 30 2-month-old female Wistar rats obtained from the animal colony in the Laboratory of Experimental Neuropathology (Institute of Zoology and Biomedical Research, Jagiellonian University, Krakow). All procedures were carried out in accordance with the permission no. 122/2015 of the First Local Ethical Committee. Before the experiment, all the animals were fed with a normal diet (ND) for rodents (Morawski Labofeed H Standard).

To control fertilization, female rats were placed in one cage with males for 8 h during the night. In the morning, every sperm-positive female was placed in a separate cage for the period of pregnancy.

**Fig. 1:**
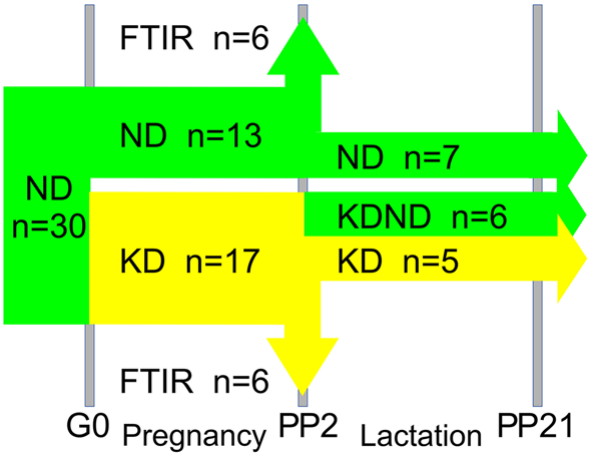
The scheme of dietary treatments of females during pregnancy and lactation periods with their numbers (*n*) in successively selected groups. Maternal blood parameters, body mass and food intake were measured on the day of fertilization (G0), on gestational days 4, 15, 20 and on the second day postpartum (PP2). On PP2, in six previously KD-fed females, the normal diet was introduced. In addition, on PP2, six ND- and six KD-fed females were subjected to perfusion-fixation and their brain processed for FTIR analysis. Neurodevelopmental reflex tests on pups were performed during the lactation period, i.e., up till day 21 postpartum (PP21). Abbreviations: ND, KD, females on normal and ketogenic diet, respectively, KDND, females initially fed with KD, which on PP2 was replaced with ND. Periods of ND and KD application are indicated in green or yellow, respectively

On the day of sperm positivity (gestational day 0, G0), the pregnant females were randomly divided into two groups. The first group (NDs, *n* = 13) remained on the normal diet while the ketogenic diet (KD, ssnif® EF R/M with 80% Fat, www.ssniff.com) was introduced in the second group (KDs, *n* = 17, Fig. 1). Table 1 shows the content of ketogenic and normal diets.

**Table 1:** The content of main nutrients (% of the dry mass) of normal and ketogenic diet

Nutrient	Ketogenic diet (KD)	Ketogenic diet (KD)
Lipids	10	79
Carbohydrates	60	1
Proteins	30	8
Others	0	12

The food consumed was weighed 3 times a week to monitor the food intake and the females were weighed on G0 and 4, 15 and 20 days after conception (G4, G15 and G20, respectively). In six females from each ND- and KD-fed group, glucose and ketone bodies (BHB—betahydroxybutyrate) blood levels were measured using an Optium Xido glucometer (Abbott Diabetes Care Ltd. Oxon, UK).

According to the Reduction Principle of 3R Rule, 2 days postpartum (PP2)*,* 12 females, previously examined for glucose and BHB blood levels, assigned for Fourier transform infrared microspectroscopy (FTIR) measurements, were weighed and perfused with physiological saline of high analytical purity. Their brains were immediately excised from the skulls, weighed and snap-frozen in liquid nitrogen. Pups of the perfused females were included in recordings of the litter size, sexual proportion and body mass on P2.

Remaining females were kept with their pups up till they were weaned on day 21 after delivery (P21). Among them, seven females from NDs and five from KDs were continuously fed with the same, respective diets. However, on PP2, in six initially KD-fed females, the diet was replaced with the normal one and continued up till the weaning time. This dietary treatment is indicated here as KDND and the animal group fed in this way as KDNDs. Figure 1 shows the scheme of dietary treatments.

### FTIR microscopy, biochemical and PCA analysis

For the topographic biochemical analysis of the whole brain slices and the hippocampal formation areas, Fourier transform infrared microspectroscopy (FTIR) was applied. The measurements were carried out in the Department of Medical Physics and Biophysics (Faculty of Physics and Applied Computer Science, AGH University of Science and Technology, Krakow) using FTIR microscope Nicolet iN10 MX (Thermo Fisher Scientific). The tissue samples placed on the glasses made of CaF_2_ were examined in the transmission mode. The absorption spectra were recorded using the linear array of 16 MCT-A detectors. They were measured for the wavenumber range of 900–4000 cm^−1^ with the resolution of 8 cm^−1^ accumulating 32 scans per each single spectrum. The spatial resolution of the measurements was around 25 μm in both directions.

The data acquisition and the topographic analysis were performed using OMNIC Picta software (version 1.5.141). To determine the distributions of compounds containing carbonyl groups (including ketone bodies), chemical maps for the band at the wavenumber of 1740 cm^−1^ were used. In turn, spectral region 1360–1480 cm^−1^, characteristic for bending vibrations of methyl and methylene groups was applied for imaging the distribution of lipids, cholesterol and/or its esters (Chwiej et al. 2015; Drozdz et al. 2020). The chemical mapping was carried out on the unprocessed spectra by displaying in two dimension the area of particular absorption band or massive. For calculations of the integrated band areas, the trapezoidal baseline correction was used and the background was taken at the two extreme frequency values of the peak/massif.

**Fig. 2:**
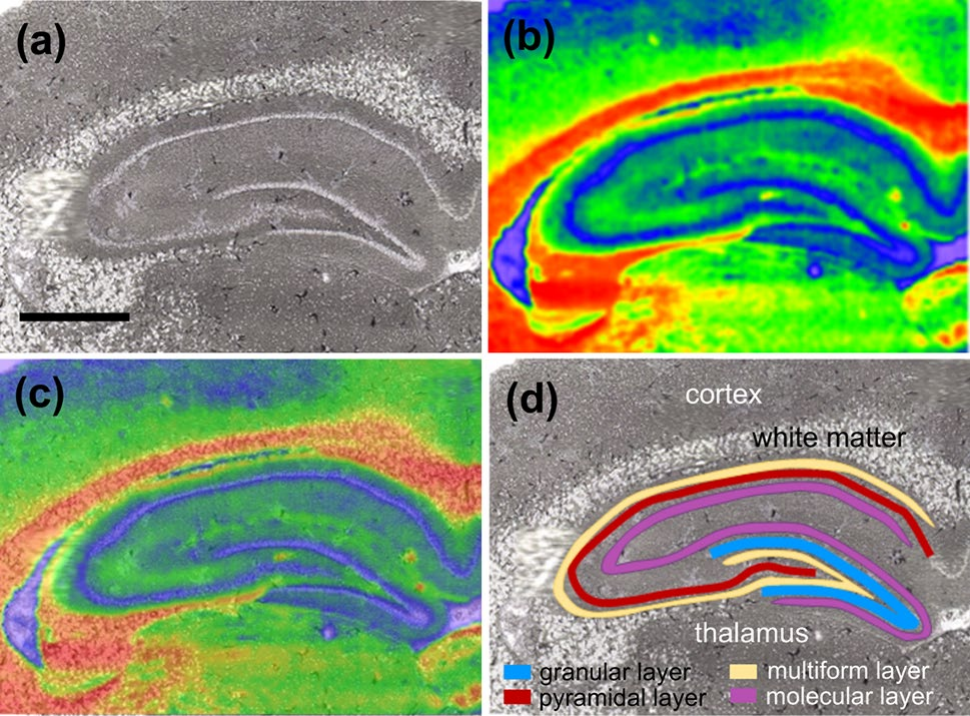
**a** Example of the examined area from an unstained brain section containing the hippocampal formation, **b** the chemical map for the area shown in part a obtained for the absorption band at 1740 cm^−1^, **c** 2D blend of pictures (**a**) and (**b**), **d** localization of pixels belonging to brain areas/cellular layers subjected to quantitative comparisons

To quantitatively compare the intensity of the examined absorption bands (1740 cm^−1^ and 1360–1480 cm^−1^) between the ketogenic- and normal diet-fed rats, for each animal their average intensities were calculated within the cortex, white matter and four main cellular layers of the hippocampal formation, namely granular, pyramidal, multiform and molecular layers (Fig. 2). To extract the pixels belonging to the particular brain areas or cellular layers, the chemical maps were imposed on the microscopic pictures obtained for each examined sample (Fig. 2c). The pixels belonging to the examined brain areas were chosen with avoiding the points localized in the border of the layers (Fig. 2d). The mean values of the biochemical parameters were calculated for 100 randomly chosen points from the examined areas/cellular layers.

Afterwards, the Mann–Whitney *U* test was applied for evaluation of statistical significance of the differences observed between the two groups. The obtained results were shown in the box-and-whiskers plots comparing the median, minimal and maximal values of the mentioned biochemical parameters in each group.

In addition, principal component analysis (PCA) was used in the study to verify if the examined brain areas/cellular layers of ketogenic- and normal diet-fed female rats can be distinguished based on their infrared absorption spectra. In other words, it was used to check if possible spectral differences between the two examined animal groups are statistically relevant and allow to categorize animals to independent clusters. The PCA was done with the use of the Origin Pro software (version 2020b) on the second derivative spectra which were previously subjected to the atmospheric and the baseline correction as well as vector normalization.

### Neurodevelopment reflex tests (Table 2)

**Table 2:** Neurodevelopmental tests, postnatal days of their introduction with positive and negative criteria

Reflex test	Postnatal day of introduction	Positive results	Examples of negatively assessed responses
Forelimb grasping	3	Two forepaws grasp rod	One forepaw grasp rod
Hindlimb grasping	3	Two hind paws grasp rod	One hind paws grasp rod
Righting	3	Full righting in 15 s	No perfect righting
Hindlimb placing	4	Bending of both stimulated hind paws	Bending of one of stimulated paws
Cliff avoidance	4	Movement away from the cliff edge	One forepaw on the cliff edge
Gait	6	At least both forepaws beyond the circle rim in 30 s	One forepaw beyond the circle rim
Auditory startle	10	Flexing body after stimulus	No response
Eye opening	12	Both fully open eyes	None or one open eye
Posture	13	Abdomen above the ground and vertically oriented limbs during movement	No rising the abdomen above the ground and paws spread wide apart
Accelerated righting	14	Full body rotation and successful fall on four legs	No successful fall on four legs

The earliest neurodevelopmental examinations begun on P3 from the following three tests:

Forelimb grasping—by applying a blunt rod to the forelimbs, the grasping reflex should be observed. A rat received a point for each forelimb. The test was scored positively when 2 points were received in two consecutive days.

Hindlimb grasping—was scored as in the previous test but concerning the hind paws.

Righting—a rotation test in which the rat was placed on its back and the time after it regained the normal position was measured. For the complete rotation occurring within 15 s, the rat was given 2 points but 1 point when the rotation was incomplete. The test was repeated until 2 points were received in two consecutive days.

From P4, more neurodevelopmental tests were applied, such as:

Hind limb placing—the rat was held upright and the upper side of the paw was rubbed against the edge of a cliff. The correct result was when the paw bended in response and a point was received for each hind paw. The test was considered passed when 2 points were received in two consecutive days.

Cliff avoidance—a pup was placed on the cliff edge and observed during 15 s. If it moved away but its paws remained on the edge, 1 point was given. If the pup moved away completely, 2 points were given. The result was negative when the pup did not move or fall from the cliff edge. The test was scored when two points were obtained within two subsequent days. A necessary assistance prevented the pups from injurious falls.

On P6, the gait test was added in which a rat placed in the center of a 15 cm diameter paper disc was expected to leave it within 30 s. The test was successfully completed when, in two consecutive days, the rat was able to cross the disc rim with, at least, both forepaws. Each rat was protected from cooling with a thick layer of polystyrene placed under the disc.

On P10, the auditory startle reflex test was introduced, where a rat, isolated from other animals, was exposed to a sharp auditory stimulus from above its head to evoke flexing its body in response. The test was passed positively when the response was repeated on two consecutive days.

From P12, eye opening was monitored to record the day when both eyes were fully open.

From P13, the rat posture during movement was observed. The rat ability to keep its abdomen above the ground with vertically oriented limbs during movements was scored positively.

Finally, the accelerated righting test was introduced on P14. Rats were tested for the ability to body rotation after they were kept upside down then dropped 30 cm above the ground. When the juvenile rats made a half turn and fell on its side, 1 point was given. The rats received 2 points when they fell on their four paws. No point was given after falling on the dorsal side. A soft floor was used in the test to protect animals from damages. A heating lamp helped to reduce the risk of hypothermia of the pups.

### Statistical analyses

The normality of data was evaluated with the Shapiro–Wilk and Levene tests. Because of the normal data distribution, diet intake and blood parameters were compared between groups using Student’s t tests for independent samples. Because of non-Gaussian distribution, the data for neurodevelopmental reflexes and offspring number or body mass were tested using the Mann–Whitney *U* test. The level of statistical significance was set at 0.05. Statistical analyses were performed with Statistica 10 (Statsoft).

## Results

### Diet intake in pregnant females

**Fig. 3:**
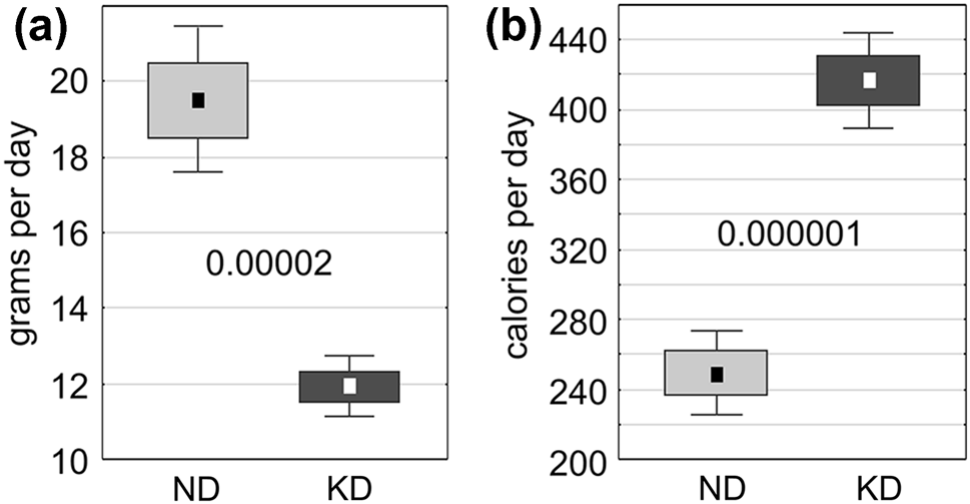
Food (**a**) and caloric intake (**b**) per day during the course of gestation in groups on the normal (ND, white boxes) or ketogenic diets (KD, black boxes), respectively. The results are presented as means (± SEM and SD) together with decimal indexes of statistical significance (Student’s t test)

**Fig. 4:**
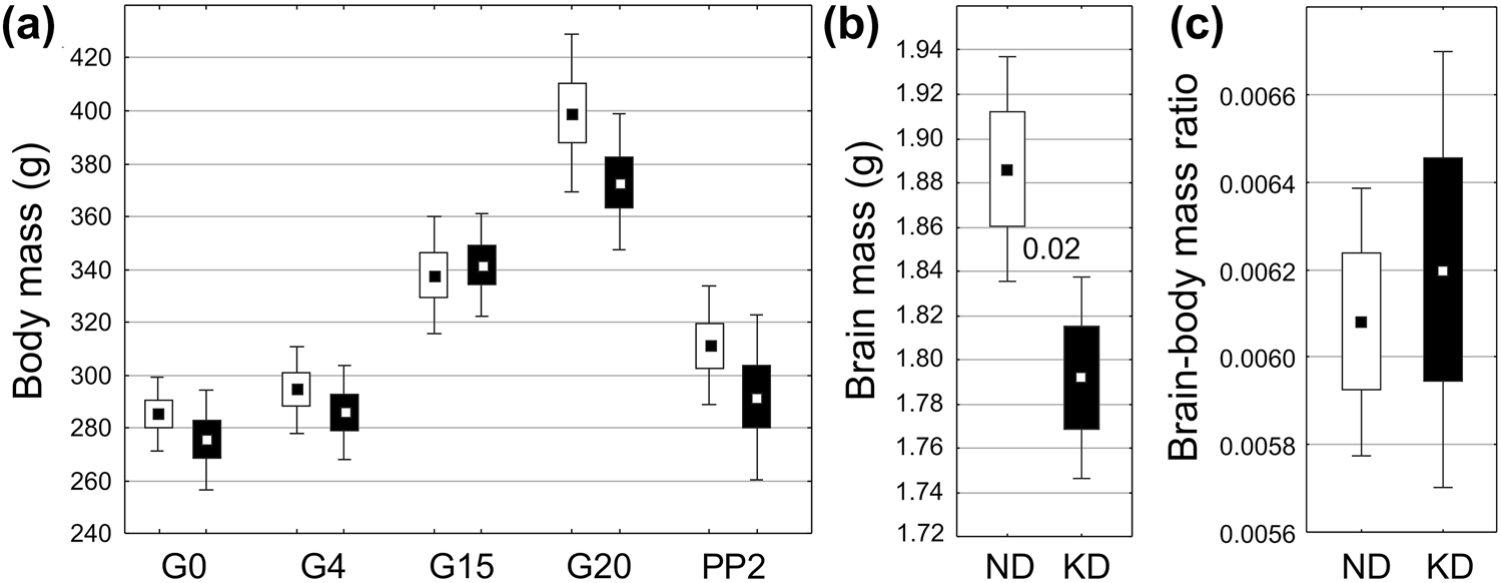
Changes in the body mass (**a**) brain mass (**b**) and in the brain–body ratio (**c**) in females on the normal (ND, white boxes) or ketogenic diets (KD, black boxes), respectively. The results are presented as means (± SEM and SD) together with a decimal index of statistical significance (Student’s t test). Abbreviations: G0, G4, G15, G20—gestational days; PP2—day 2 postpartum

In the group of KD-fed females, the daily food intake was significantly lower than in those fed with ND (*p* < 0.00002, Fig. 3a), but this relation was quite opposite in terms of the caloric intake, which was significantly higher in KD-fed females (*p* < 0.000001, Fig. 3b). Interestingly, the average body masses in these two female groups recorded at four stages of pregnancy and on the second day postpartum (PP2) did not reveal any significant difference (Fig. 4a). On PP2, however, when six brains were collected from each of the two female groups for biochemical analysis, a significant decrease in the brain mass was revealed in KD-fed females (Fig. 4b; *p* < 0.02). This difference, however, was not reflected by a corresponding significant difference between the brain-to-body mass ratios (Fig. 4c). The problem of the long-term effects of KD and of their possible reversibility in pregnant females could be a reasonable subject for further research.

### Blood parameters in pregnant females

**Fig. 5:**
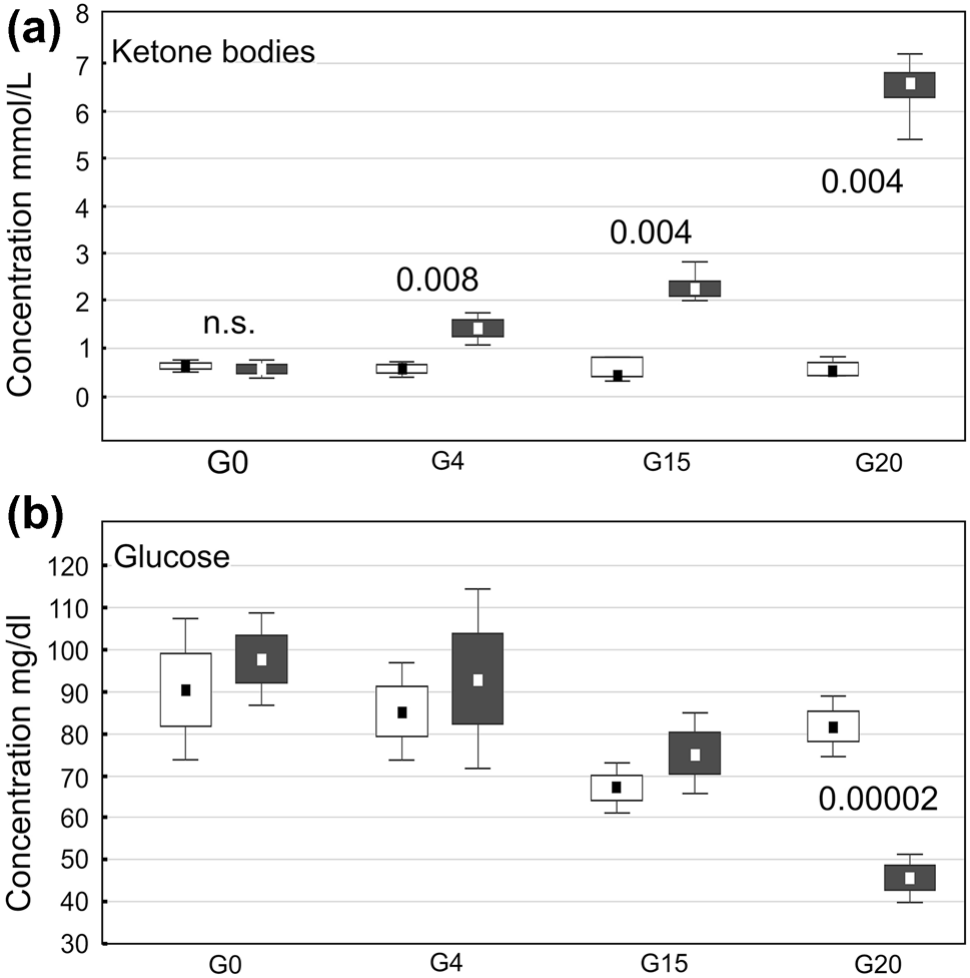
Blood levels of ketone bodies (**a**) and of glucose (**b**) measured on gestational days 0, 4, 15 and 20 (G0, G4, G15 and G20, respectively) in ND- and KD-fed females (white and black boxes, respectively). The results are shown as medians with Q25, Q75, minima and maxima (small squares, boxes and whiskers, respectively). Decimal indexes show statistical significance of differences between adjacent graphs (nonparametric Mann–Whitney *U* test)

The blood level of BHB was significantly higher in KD-fed pregnant rats on G0, G4, G15 and G20 (*p* < 0.008, *p* < 0.004, *p* < 0.004, respectively) than that in those fed with ND (Fig. 5a). The glucose blood level was similar on G0, G4 and G15 in both groups, however, on G20 it was higher in ND. (*p* < 0.00002, Fig. 5b).

### Brain biochemical composition

**Fig. 6:**
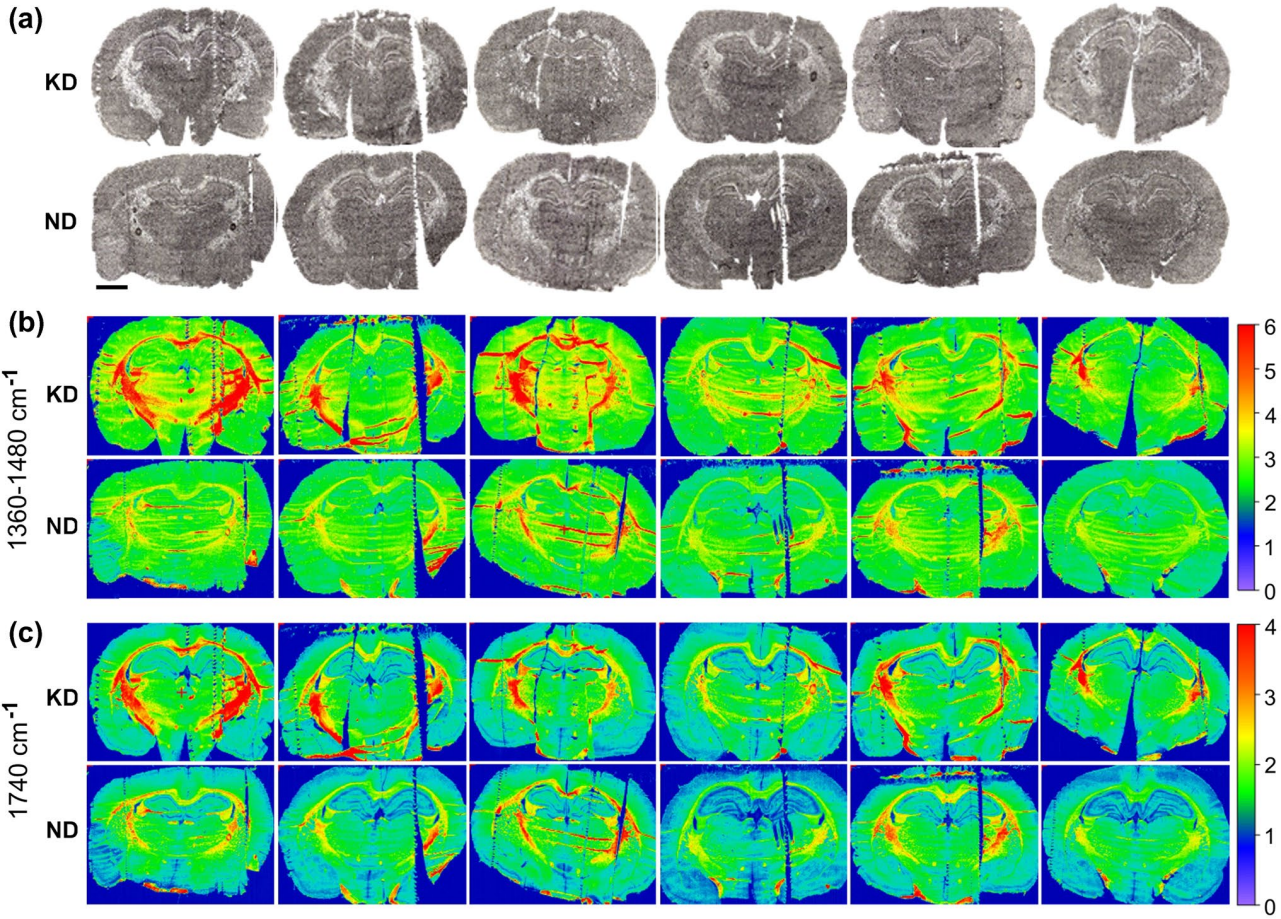
Anatomical images and chemical maps of brains taken from female rats fed with ketogenic (KD) and normal diet (ND): **a**microscopic views of unstained brains sections corresponding to chemical maps showing results of FTIR analysis and presented in parts ** b** and **c** of the figure; **b** and **c** the chemical maps showing the distribution of intensity of the 1360–1480 cm ^−1^ massif and 1740 cm^−1^ band, respectively. Color scales on the right represent the range of changes in the particular band/massif intensity. The bar shows 2.5 mm

**Fig. 7:**
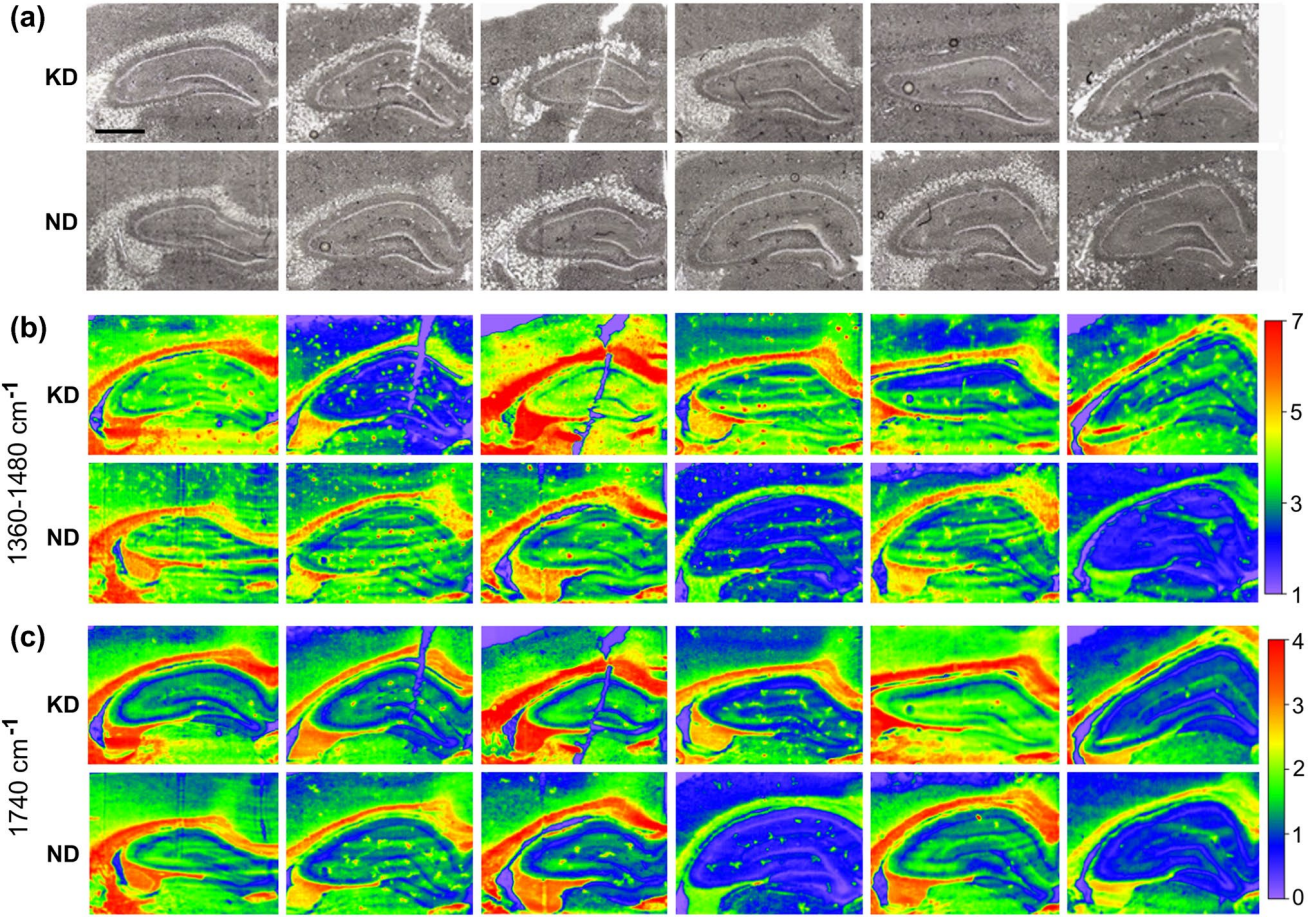
Anatomical images and chemical maps of the hippocampal formation taken from female rats fed with ketogenic (KD) and normal diet (ND): **a-**microscopic views of hippocampal formations corresponding to chemical maps presented in parts b and c of the figure; **b** and **c-**the chemical maps showing, at higher magnification, the distribution of intensity of the 1360–1480 cm^−1^ massif and 1740 cm^−1^ band, respectively. Color scales on the right represent the range of changes in the particular band/massif intensity. The bar shows 1.0 mm

In Fig. 6, the microscopic images of female rat brains were compared with the two-dimensional chemical maps presenting the distribution of 1360–1480 cm^−1^ and 1740 cm^−1^ bands intensities. As one can see, in case of KD-fed female rats, the surface of white matter characterized by higher accumulation of both absorption bands appears larger. Such relationship is not visible for the hippocampal formation what one can see in the Fig. 7.

**Fig. 8:**
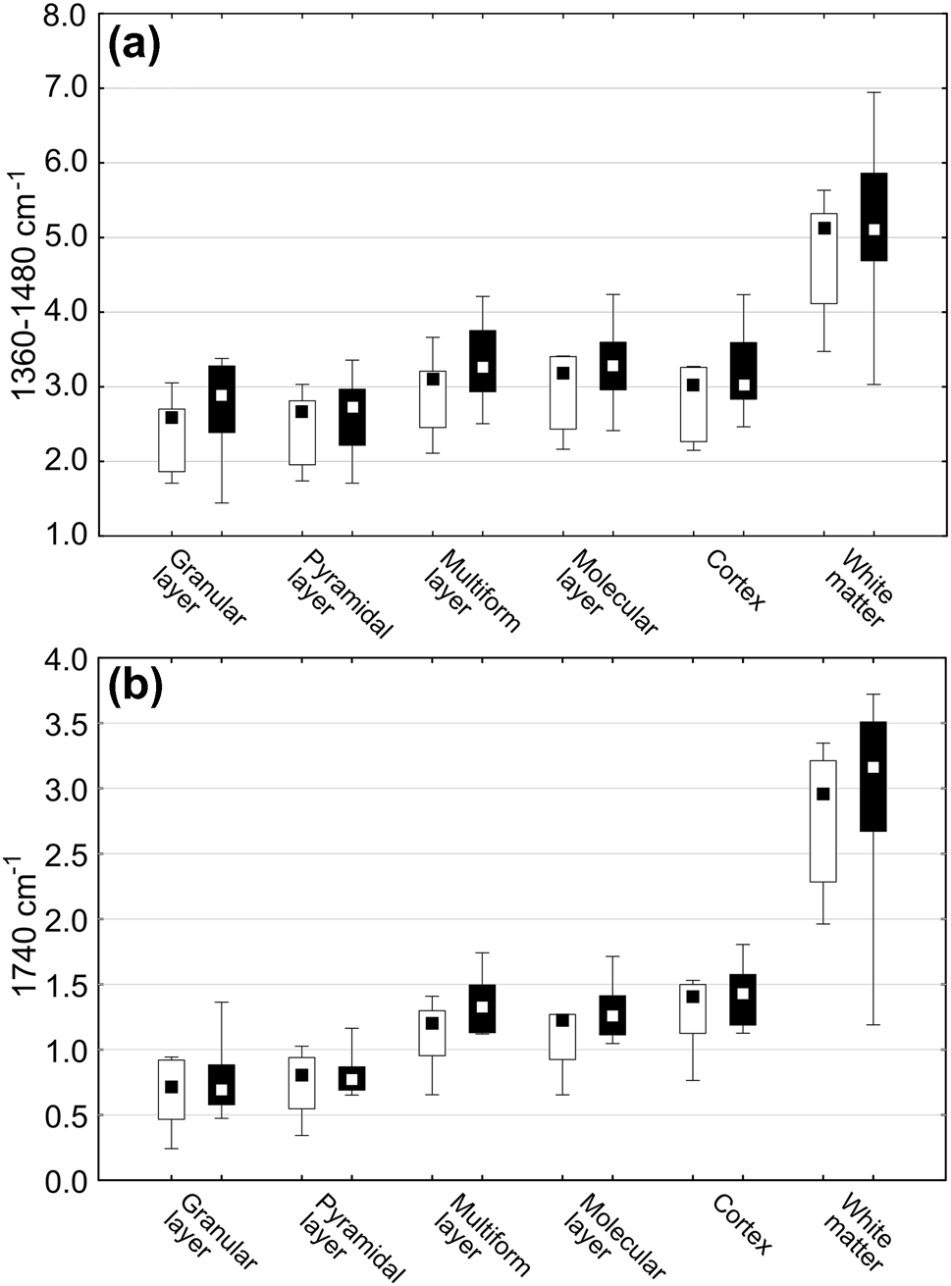
The spread of the absolute intensity of 1360–1480 cm^−1^ (**a**) and 1740 cm^−1^ (**b**) absorption bands recorded within the granular, pyramidal, multiform and molecular layers of the hippocampal formation and the cerebral cortex a underlying white matter. The regions are indicated under the abscissa. Medians, interquartile spans and minimal–maximal values are marked as little square, boxes and whiskers, respectively. No statistically significant differences were detected (nonparametric Mann–Whitney *U* test). White and black boxes represent groups of ND- and KD-fed females, respectively

Mann–Whitney’s *U* test used to verify statistical significance of differences in the level of 1740 cm^−1^ and 1360–1480 cm^−1^ absorption bands intensities between KD and ND groups did not confirm the existence of statistically relevant anomalies for any of the examined areas and the cellular layers (Fig. 8). However, the intensity of 1740 cm^−1^ absorption band tended to be higher in matter of females fed the ketogenic diet.

**Fig. 9:**
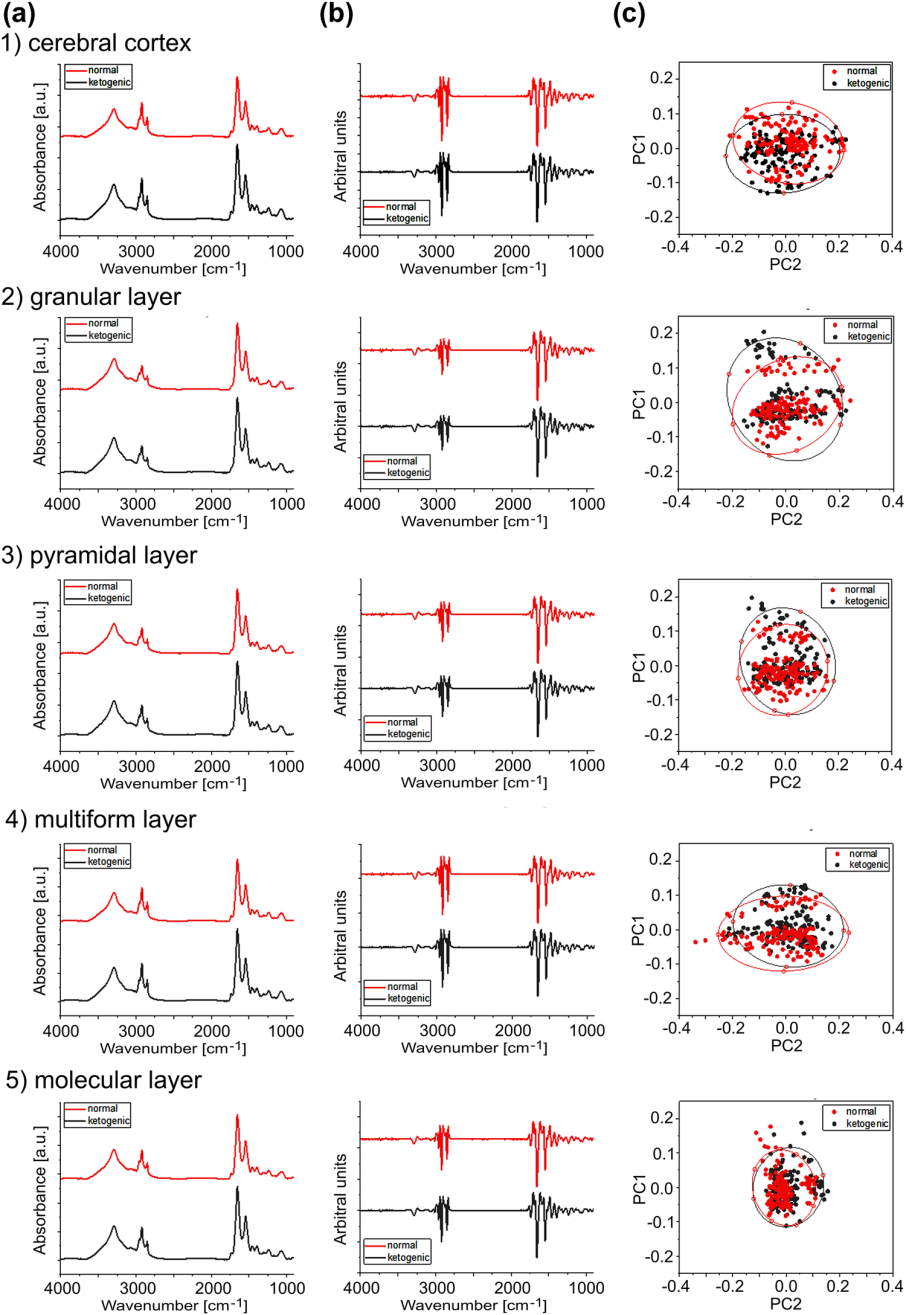
Average spectra, subjected to atmospheric and baseline corrections (column **a**) and second derivative spectra (column **b**) obtained from the cerebral cortex (1) and granular (2), pyramidal (3), multiform (4) and molecular (5) layers of the hippocampal formation of ND- and KD-fed females (red and black traces, respectively). The results of PCA performed on the second derivative of vector-normalized spectra, separately for each examined region or cellular layer are shown in the column (**c**)

The carried out PCA showed that the infrared absorption spectra recorded for ketogenic and normal diet-fed animals did not differ significantly (Fig. 9). As the analysis took into account all the components of the recorded infrared spectra, its results suggest that there are no global biochemical differences between brain tissue taken from female rats fed with the normal and ketogenic diet. Such a result is also in agreement with the quantitative results obtained for the examined absorption bands which did not show statistically relevant differences in intensities.

### Numbers, body mass and general appearance of offsprings of females fed with different diets

The numbers of newborn rats in particular litters and their sexual proportions did not differ between the examined groups (data not shown), however, very strong effects of different dietary treatments were detected.

**Fig. 10:**
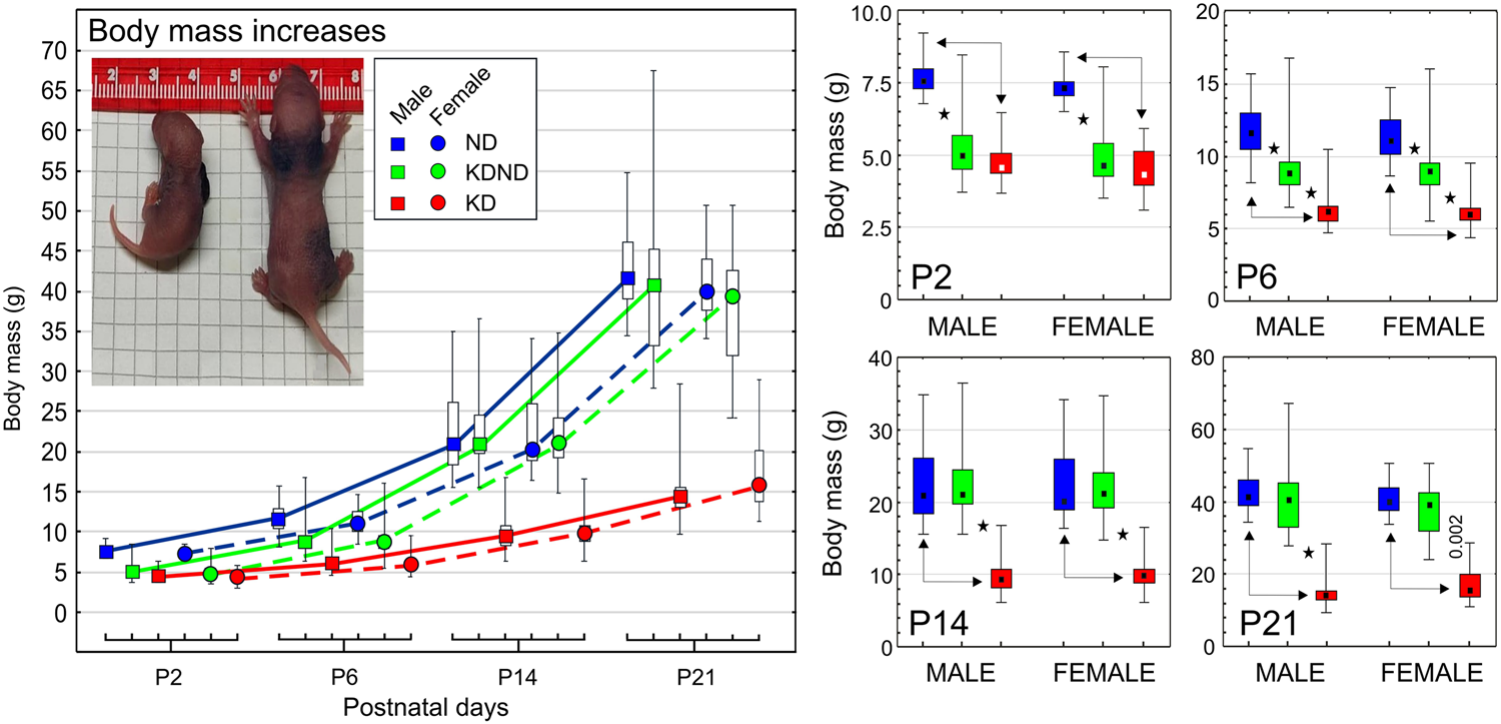
Developmental increases in the body mass of rat pups. An inserted photograph shows representative examples of 2-day-old pups born by KD- (left) and ND-fed (right) females. Details of intergroup relations on P2, P6, P14 and P21 are shown in diagrams with respective labels. The pups from females fed exclusively with the normal (ND) and ketogenic (KD) diets, and pups originating from females initially receiving KD, which on day 2 postpartum was replaced with ND (KDND), are indicated according to the inserted legend. The results are shown as medians with Q25, Q75, minima and maxima (small squares, boxes and whiskers, respectively). Asterisks and double-headed arrows indicate intergroup differences statistically significant at levels not lower than 10^–6^, except for that significant at *p* < 0.002 (nonparametric Mann–Whitney *U* test)

Offsprings of females that were fed KD during their pregnancy and up till the 2nd day postpartum showed highly significant reductions of the body mass compared to their counterparts from ND-fed females. When a group of females, originally fed with KD, began to receive ND on PP2 (from that day this group was indicated as KDNDs), their offspring began to restore the body mass very quickly. It fact, on P6, it was still significantly lower than that in the group fed with ND alone but already significantly higher than in the group which was continuously fed with KD. From P14, the offsprings of KDND-fed females showed complete restoration of their body mass in relation to pups continuously depending on ND and disappearance of previous difference between them. Consequently, the average body mass of pups from these groups were significantly higher than that observed in pups depending on KD alone. These relations remained up till P21. Intergroup differences showed statistical significance, at least at the level of p = 10^–6^, except for p < 0.002 for the difference detected on P21 between female pups from KDND- and KD-fed females, a drastic decrease in the body size of the KD-fed pups could also be observed (Fig. 10).

### Neurodevelopment reflex tests (Table 2, Fig. 11)

**Fig. 11:**
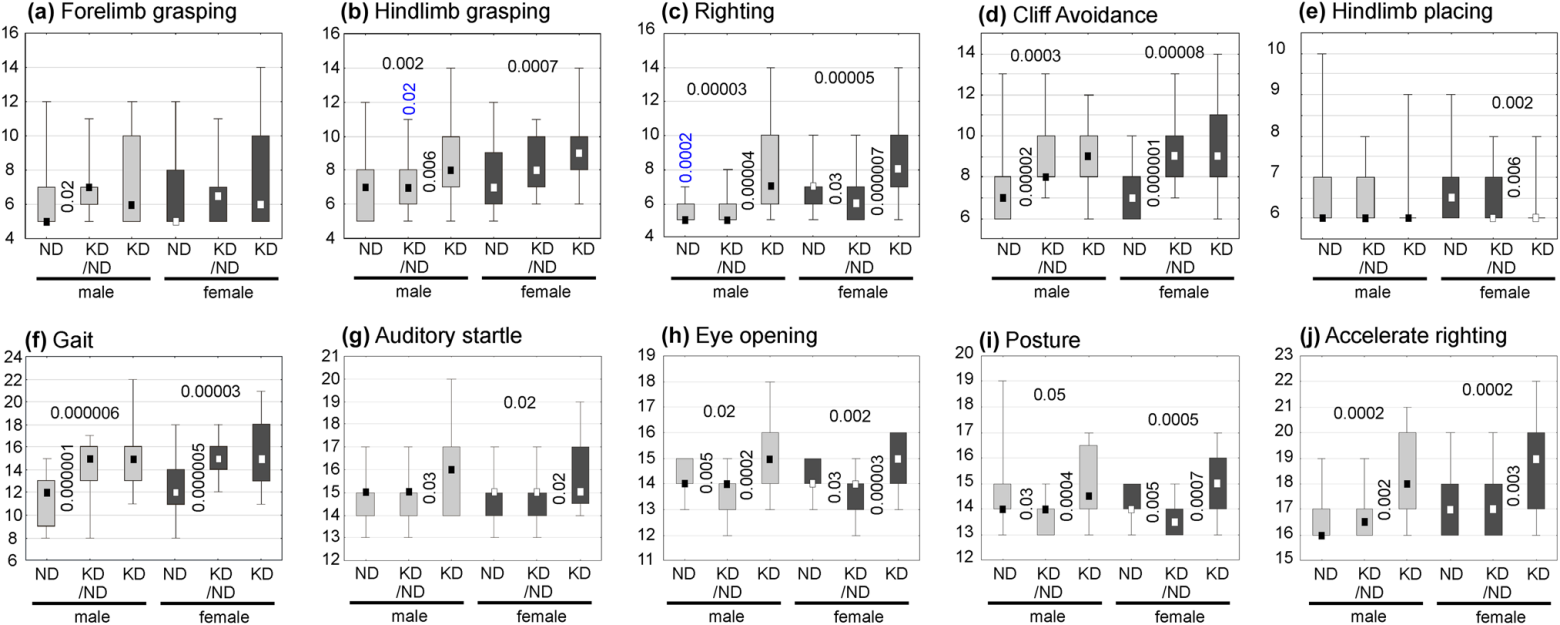
Results of neurodevelopmental tests related to days of postnatal development shown separately for male and female pups. It is noteworthy that in these graphs higher values indicate greater developmental delays in obtaining positive results. Abbreviations: offsprings of females fed continuously with normal (ND, grey boxes) or ketogenic (KD, black boxes) diets during their pregnancy and lactation periods. Offspring of females fed with KD during pregnancy and up till the 2nd postnatal day and, thereafter, with ND (symbolized as KDND) are represented by grey boxes. The results are shown as medians (small squares) with Q25 and Q75 (boxes), and minima and maxima (whiskers). Vertically oriented decimal indexes show levels of statistical significance (Mann–Whitney *U* test) between two animal groups represented by adjacent graphs. Horizontally oriented indexes characterize differences between offsprings of ND- and KD-fed females. Vertical indexes in blue, located over whiskers in (b) and (c), refer to statistical significance of differences between corresponding male and female groups

(a) Forelimb grasping

A delay in acquisition of the forelimb grasping (*p* < 0.02) was detected in KDNDs in relation to NDs without any other significant difference (Fig. 11a).

(b) Hindlimb grasping

In males, a positive completion of this test was significantly delayed in all KDs compared to NDs (*p* < 0.002) and KDNDs (*p* < 0.006, Fig. 11b). In females, the delay was significant only in KDs (*p* < 0.0007). In addition, results of KDND-fed males were better than of their female counterparts (*p* < 0.02).

(c) Righting

The righting test detected significant decreases ability in all KDs, in relation to NDs (*p* < 0.00003 and *p* < 0.00005, for males and females, respectively, Fig. 11c) and in relation do KDNDs (*p* < 0.00004 and *p* < 0.000007, for males and females). KDND females showed a slight improvement in relation to corresponding NDs (*p* < 0.03). In ND-fed rats, the test performance was earlier in males than in females (*p* < 0.0002).

(d) Cliff avoidance

Similar delays, versus NDs, in the development of cliff avoidance reflex was displayed by males (*p* < 0.0003 and *p* < 0.00002, for KDs and NDKDs, respectively, Fig. 11d) and by females (*p* < 0.00008 and *p* < 0.000001, for KDs and NDKDs, respectively).

(e) Hindlimb placing

This test showed no significant difference in male groups. However, the acquisition of hindlimb placing ability in KD female pups was significantly slower than in NDs (*p* < 0.002) or in KDNDs (*p* < 0.006, Fig. 11e).

(f) Gait

According to the gait test, scores of KDs and KDNDs were similar but, when compared to NDs, both groups showed functional retardation in males (*p* < 0.000006 and *p* < 0.000001, respectively, Fig. 11f) and females (*p* < 0.00003 and *p* < 0.000005).

(g) Auditory startle

A developmental slowdown in KDs was detected. In males, it was statistically significant only when compared to KDNDs (*p* < 0.03, Fig. 11g) but in females, it occurred also in relation to NDs (*p* < 0.02) and NDKDs (*p* < 0.02).

(h) Eye opening

Both male and female KDs opened their eyes later than NDs (*p* < 0.02 and *p* < 0.002, respectively, Fig. 11h) or KDNDs (males: *p* < 0.0002 and *p* < 0.00003, respectively). Interestingly, in relation to NDs, eye opening in male KDNDs was significantly accelerated, both in males (*p* < 0.005) and females (*p* < 0.03).

(i) Posture

The acquisition of the correct posture showed a similar patterns of intergroup differences as that characterizing the development of eye opening. When compared with NDs, significant delays were recorded in KDs, male (*p* < 0.05) and female (*p* < 0.0005, Fig. 11i). In KDNDs, the posture development was quicker than in NDs, also in males (*p* < 0.03) and females (p < 0.005). Consequently, respective differences between KDNDs and KDs became greater in male (*p* < 0.0004 and female (*p* < 0.0007) pups).

(j) Accelerated righting

The accelerated righting test showed a significant delay in KDs, in comparison with KDNDs (*p* < 0.002 in males and *p* < 0.003 in females, Fig. 11j) and with KDs (p < 0.0002 and* p* < 0.0002 for males and females, respectively).

## Discussion

Drug-resistant epilepsies constitute almost 30% of all epileptic cases. However, even when pharmacotherapy can be used effectively, its harmful side effects frequently exclude its application in pregnant women (Tomson 2019) and the only alternative can to be the use of a ketogenic diet. Most of the clinical research on this topic focuses on monitoring women’s health and the course of the disease. Our experiment was the first published attempt at a long-term assessment of the physiological condition of offsprings to female who were on a ketogenic diet during pregnancy. In experimental conditions, we could study the effect of the diet used, without taking into account accompanying changes caused by the development of the disease or previously used therapies. Such conditions also allowed, to a greater extent, to focus on the assessment of developmental parameters.

For the first time, our research presents a detailed description of the course of pregnancy in KD-fed rat females from the very beginning of pregnancy to the end of lactation period. It became clear that, despite the intake of high-calorie food, in which as much as 80% of energy comes from fat, their average body mass at any of the four time points of pregnancy (up till G20) was not greater than that of the ND-fed females. However, the level of BHB in their peripheral blood was significantly higher from G4 and increased up till postnatal day 21, when it reached the level of 7 mmol/l. According to the literature data (Herrera et al. 1987), ketone bodies circulating in the maternal blood easily cross the placenta. Under the conditions of a normal diet, the fetal development depends on a continuous supply of glucose and appropriate amino acids. Any insufficiency in this supply affects the metabolism in both the mother and the fetus (Barry 2018).

Our research showed that the glucose level in KD-fed females was lowered only at the last measurement point (G20), i.e., about two days before delivery, so there should rather not lead to any important disturbances. As it is known, long-term ketonemia during pregnancy can evoke metabolic encephalopathies (Al-Mudallal et al. 1995). Sussman (2013) showed that in the early stages of pregnancy, KD intensifies the heart growth (at E13.5) and reduces the growth of the pharynx, cervical spine, pons, midbrain and hypothalamus. On E17.5, she observed a reduction of the heart and thymus, and an enlargement of the cervical spine, pons, midbrain and thalamus. This discrepancies in organ growth may be due to variations in the availability of metabolic “fuel”. As revealed by Lust et al. (2003), on the 18th day of gestation in ND-fed females, a significant increase in BHB content was found in the fetal brains.

Our observations showed that KD does not affect the duration of pregnancy, and all the females, regardless of the type of diet applied, gave birth 21–22 days after conception. The number of pups in the litter also did not differ between the animal groups, and there were no changes in proportions of male and female pups. This is likely due to the fact that all pregnant females were still on ND at the time of conception. However, on P2, a significantly reduced body mass was observed in animals born by females treated with KD during their pregnancy. This difference persisted on postnatal days 6. Similar results were obtained by Mark et al. (2011) and Mendes-da-Silva et al. (2014) using a high-fat diet in pregnant females, and Soares et al. (2009) using two KD types with different fat contents. Despite the considerably lowered body mass, we did not observe any morphological anomalies in the pups born by females kept on KD during pregnancy. This early reduction in the body mass was compensated relatively quickly following, as evidenced on P14 by disappearance of differences between offsprings of KDND- and ND-fed females which is the effect of KD replacement with ND. On the contrary, a continuous application of KD resulted in a constant reduction of the body mass during the whole examined period, i.e., up to the weaning time (P21). However, this is only an indicator of compensation in terms of general somatic development, which does not have to correspond with an improvement in the functional neurological state which depends on disorders of the much more restrictive calendar of brain development.

In the present investigation, FTIR microspectroscopy was used to verify if ketogenic diet feeding of pregnant females leads to biochemical changes within their brains. Before, we have already performed a similar study (Chwiej et al. 2015), but only on 30-day-old males who were treated with KD for next 30 days. So far, such studies have not been performed on females. The topographic analysis of chemical maps presenting the distribution of 1740 cm^−1^ and 1360–1480 cm^−1^ bands specific for compounds containing carbonyl groups, lipids, cholesterol and/or its esters showed significantly increased surface of the white matter characterized by the elevated intensities of these bands. Further quantitative and statistical analysis of the obtained results did not point, however, that the accumulations of the mentioned compounds was changed within the examined brain areas and cellular layers in KD-fed females. Thus, the changes such as increased levels of lipids and carbonyl groups containing compounds observed previously in males where not confirmed in this study in females. This result is quite surprising considering that there are studies, which proved that ketone bodies easily passed through the BBB and could accumulate within the brain parenchyma and their increased availability implied a subsequent increase of the lipid synthesis (Barry 2018). In addition, the results of PCA did not show any general differences between the spectra recorded for females fed with ketogenic and normal diet in particular layers of the hippocampal formation, neocortex and white matter.

To assess how KD, low in proteins and sugars, applied in pregnant females, would affect the postnatal development of their offsprings, a series of neurodevelopmental reflexes was performed in three groups of animals whose mothers (i) were fed KD during pregnancy and lactation (KDs), (ii) were fed KD only during pregnancy and ND from the second postpartum day (KDNDs), and (iii) were fed exclusively with ND (NDs). The tests assessed some general parameters of physiological development (like eye opening and posture), reflexes (forelimb and hindlimb grasping, hindlimb placing), body rotation and auditory startle. The tests were successively introduced from P3, when it was not yet possible to observe more complex behavioral parameters. These tests, just like in human newborns, could predict developmental disorders of various origins, such as, for example, cerebral palsy (Nguyen et al. 2017).

All the neurological tests were performed separately on males and females, but the hindlimb grasping and righting tests were the only two of them which detected intersexual differences. The righting test in male pups born by ND-treated females was performed significantly earlier than in their female counterparts originating from KD-treated females. Similarly, the male pups of KDND-fed females were better in the hindlimb grasping tests. No gender-dependent differences were observed in any other test, regardless of the type of food consumed during pregnancy. It appears, therefore, reasonable that until puberty (at about P21), differences between behaviors of males and females may not be expected. Similar conclusions were presented by Sussman et al. (2015).

Interestingly, two important physiological symptoms of maturity, i.e., opening the eyes and maintaining a proper posture, were first observed in the offspring of KDND-fed females, compared to those from females which were always on ND. Similar results were observed by Nguyen (2017), in pups whose mothers received broccoli extract during pregnancy and had induced inflammation. Studies on rodents show that eye opening is correlated with changes in the GABAA receptor subunit in the visual cortex (Heinen et al. 2004). Thus, earlier eyes opening may indicate an intensification of plastic processes in the cortex supposedly evoked by the dietary changes.

In most of the performed tests (except for hindlimb placing), the maintenance of KD throughout pregnancy, as well as during the period including both pregnancy and lactation, delayed the performance of the tests in respective offsprings as compared to the progeny ND-nourished females. The most developmentally delayed was the performance of the neurological tests in animals whose mothers were fed KD not only during pregnancy, but also during lactation. In ND-fed animals, the energy from the lactate metabolism transformation is obtained only shortly after birth. Then, until the end of lactation period, the ketone bodies are the source of energy for the pups (Prins 2012), because during the neonatal period their brains are not able to efficiently use glucose as a metabolic substrate (Nehlig 1999). During this time, not only does the amount of ketones in the body increase, but also the number and activity of their transporters in the blood–brain barrier (Vannucci and Simpson 2003).

This is why the immature rat brain is able to take up and accumulate ketone bodies faster and more efficiently than the mature one (Nehlig 1999). During the period of peak ketone utilization, the brain ability to uptake ß-hydroxybutyrate (ßOHB) is sixfold greater than that in adulthood (Cremer et al. 1976). Therefore, if the lactating females are additionally fed with KD, these changes may be intensified and lead to metabolic acidosis (Sussman 2013), which reduces lactation and thus inhibits the developmental progress of the pups. This results, apart from body mass reduction, in definitely delayed muscle development and developmental brain disorders leading to worse performance of neurodevelopmental reflexes.

The prenatal period is critical to the development of the nervous system (Rice and Barone 2000) and, apart from stress or inflammation, it can be affected by malnutrition (Cusik and Georfieff 2016). The continuity of prenatal and postnatal development should also be taken into account. Thus, effects of prenatal malnutrition would occur also in brain structures that develop postnatally (Morgane et al. 2002). Following KD consumption during pregnancy, as much as 80% of energy derives from metabolized fats and as much as 70% less protein is provided than in the case of ND. Protein deficiency in the diet of pregnant females, as described by Abey (2019), delayed neurological and postural reflexes. The results of our tests confirm this observation in the offsprings of females receiving KD only during pregnancy or during both pregnancy and lactation. We also observed depleted hair in the offspring of females who were fed with KD while being pregnant and lactating. This could be due to inadequate development of the hair follicles (Guo and Katta 2017) resulting from protein deficiency. Another reason for the delays in the tests of neurodevelopmental reflexes shown by the offspring of KD-fed females might be a reduction of forebrain vascularization in animals on a protein-deficient diet (Bennis-Taleb et al. 1999) which can be preserved up till adulthood.

During pregnancy, the protein-poor diet may also decrease expression of brain neurotrophins in the rat offspring including that of BDNF which, in turn, affects neuro- or synaptogenesis (Marwarha et al. 2017) resulting in worse performance of neurodevelopmental tests.

The results of seven from of the ten neurological tests applied in the present study clearly indicate that the ketogenic diet withdrawal may lead not only to a general, unspecific restitution of somatic condition, represented here by the compensatory regain of reduced body mass, but may also result in functional restitution of the nervous system, although not to the full extent. However, our study has considered developmental period up to the weaning time, so a further progress in the functional amelioration up till the complete adulthood cannot be excluded. Thus, the first and most important task for further research in this field is to detect, if not the specific mechanisms underlying these changes, then at least their structural correlates.

## Conclusions

Taking into account the presented results, one should consider the rationale of KD application in pregnant women suffering from epilepsy. All over the world, antiepileptic drug therapies include as many as 15 million women in their reproductive age and bring the risk of serious birth defects and adverse effects on the neurocognitive and behavioral development of newborn babies. However, uncontrolled seizures can also be harmful not only to the pregnant woman but also to the fetus (Tomson 2019). Therefore, other measures to prevent seizures during pregnancy, including KD, are often considered. However, case reports are related rather to the efficacy of dietary regimen in suppressing seizures in pregnant women but not on its effects on their babies. Seizure amelioration occurs and is accompanied by increases in blood lipids, which, however, return to the norm after delivery. Children of these mothers are born at term with a reduced body mass (van der Louw et al. 2016), but their further fate has not been studied. Rizzo et al. (1995) monitored infants whose pregnant mothers suffering from diabetes had highly increased levels of ketones. The results of motor skills tests of these children were inversely related to the maternal β-hydroxybutyric acid content in blood during pregnancy, and their IQ was inversely correlated with the maternal blood ketone content.

Considering the above-mentioned results, we intend to continue our research on older, 30- and 60-day-old rats born to females fed KD during pregnancy. We are going to assess whether the neonatal changes observed in the present study will be worsen or compensated during further development. In addition to behavioral and histological studies, we would also like to focus on changes in the molecular composition of the nervous tissue. Such an interdisciplinary approach will improve understanding of the mechanisms underlying the already documented action of the ketogenic diet.

## Data Availability

My manuscript has no associated data.

## Declarations

### Human and animal participants right statement

This article does not contain any studies involving human participants that were performed by any of the authors.
