# Correction to: Ketogenic diet impairs neurological development of neonatal rats and affects biochemical composition of maternal brains: evidence of functional recovery in pups

**DOI:** 10.1007/s00429-022-02500-2

**Published:** 2022-04-29

**Authors:** Wojciech Kosiek, Zuzanna Rauk, Piotr Szulc, Anna Cichy, Marzena Rugieł, Joanna Chwiej, Krzysztof Janeczko, Zuzanna Setkowicz

**Affiliations:** 1 Laboratory of Experimental Neuropathology, Institute of Zoology and Biomedical Research, Faculty of Biology, Gronostajowa 9, Krakow, 30-387 Poland; 2 Faculty of Biochemistry, Biophysics and Biotechnology, Gronostajowa 7, Krakow, 30-387 Poland; 3 Faculty of Physics and Applied Computer Science, AGH, University of Science and Technology, Krakow, 30-059 Poland

## Correction to: Brain Structure and Function (2022) 227:1099–1113 10.1007/s00429-021-02450-1

In the original version of this article, the given and family names of the authors below were incorrectly structured.

The correct author names should read as

Wojciech Kosiek, Zuzanna Rauk, Piotr Szulc, Anna Cichy, Marzena Rugieł, Joanna Chwiej, Krzysztof Janeczko, Zuzanna Setkowicz

The original article has been corrected.

